# A Dual-Modified Chitosan-Derived Silica Composite Aerogel with Simultaneous Improvement of Mechanical, Flame Retardancy, and Thermal Insulation Properties

**DOI:** 10.3390/polym17233162

**Published:** 2025-11-27

**Authors:** Sicong Zhou, Ying Hou, Guifeng Xiang, Chuang Hu, Baisong Hu, Yingxi Ji, Wei Zhang, Shaofeng Zhang

**Affiliations:** 1 School of Chemical Engineering and Technology, Hebei University of Technology, Tianjin 300130, China; 2 Chongqing Innovation Center, Beijing Institute of Technology, Chongqing 401120, China; 3 State Key Laboratory of Explosion Science and Safety Protection, Beijing Institute of Technology, Beijing 100081, China; 4 Hubei Aerospace Jianghe Chemical Co., Ltd., Yichang 444200, China

**Keywords:** dual-modified aerogel, intumescent flame retardant, mechanical properties, thermal insulation, fire warning

## Abstract

Energy-efficient buildings require materials with low thermal conductivity, and high fire resistance and mechanical properties. Traditional chitosan aerogel is limited by its poor fire resistance and silica aerogel is either brittle or displays a high thermal conductivity. Herein, we report a dual-modified aerogel with high mechanical properties, low thermal conductivity, and excellent fire resistance. Briefly, the chitosan-derived silica aerogel (CA) was first fabricated, followed by loading varying contents of phytate-piperazine (PPA) intumescent flame-retardant. This well-designed aerogel (CA/PPA) combines with the high mechanical strength of chitosan aerogel, the excellent fire resistance function of silica aerogel, and the intumescent flame-retardant performance of PPA. The as-created CA/PPA-2.0 exhibited enhanced mechanical properties, as evidenced by the fact that its compressive strength rose from 1.3 ± 0.10 to 2.9 ± 0.23 MPa at 90.0% strain compared to that of neat chitosan aerogel. Additionally, compared to CA, the CA/PPA aerogel with 2.0 wt% PPA exhibits significant fire safety performance. For instance, the peak heat release rate, total heat release, maximum average rate of heat emission, and thermal conductivity were reduced by 62.1%, 55.6%, 48.4%, and 12.5%, respectively. In addition, the CA/PPA-2.0 composite aerogel also exhibits a fire-alarm performance under flame attack. This work introduces a feasible strategy to produce high-performance aerogels with promising applications in construction, aerospace, and thermal insulation.

## 1. Introduction

Energy-efficient buildings require materials with a low thermal conductivity and high fire resistance and mechanical properties [1,2,3]. As global demands for energy efficiency, environmental sustainability, and advanced material innovation become increasingly urgent, silica (SA) aerogels have attracted great interest [4,5]. Traditional SA aerogels have a three-dimensional skeleton structure formed by the accumulation of ultrafine nanoparticles with remarkable properties, such as low bulk density and thermal conductivity [4]. During use, it is prone to structural collapse, posing potential safety hazards [6].

The loading of powder, polymer, and fibers for enhancing the mechanical properties have been widely explored [7]. These hybrid aerogels have typical features, such as high thermal insulation and high mechanical strength. Most polymers have been used to improve the mechanical properties of SA aerogels. These polymers are composed of macromolecular substances, mainly including epoxides, polystyrene, and polysaccharide polymers [8,9]. For instance, Kantor et al. [8] have developed a silica–polyimide hybrid aerogel, whose exceptional characteristics have created opportunities for research in building insulation applications. Similarly, Chen et al. [9] proposed an efficient preparation method for silica–cellulose composite aerogels.

Chitosan (CS), a naturally occurring cationic polysaccharide, demonstrates exceptional biocompatibility, intrinsic bactericidal and hemostatic properties, and low production costs, making it an ideal candidate for the fabrication of hybrid CS–SA cross-linked aerogels [10,11]. Additionally, CS contains amino (-NH_2_) and hydroxyl (-OH) functional groups that can undergo condensation reactions with silicon precursors, leading to the formation of stable covalent bonds (Si-O-C and Si-O-N). While hybrid modifications improve the structural stability and mechanical properties of SA aerogels, the incorporation of organic moieties concurrently introduces significant fire hazards [12,13]. The inherent fire risk associated with hybrid chitosan–silica aerogels considerably limits their widespread application as high-performance thermal insulation materials under practical conditions. A viable strategy for mitigating the fire hazards of hybrid aerogels is the incorporation of flame-retardant additives into the matrix [12,13].

Intumescent flame retardant (IFR) has garnered significant attention due to its advantages, including high efficiency, halogen-free composition, and environmental friendliness [14,15]. In IFR systems, acid catalysts, blowing agents, and charring agents are incorporated. These components interact with one another during the combustion process to produce a protective residual char [11,16]. Due to the high nitrogen and phosphorus content of ammonium polyphosphate (APP), it is commonly used as the acid source and blowing agent in conventional IFR. However, the utilization of APP is significantly limited, primarily due to its inadequate water resistance and weakened compatibility with polymer chains. Additionally, the IFR compounding process is often time-consuming [17,18]. A novel IFR that integrates the functions of an acid catalyst, blowing agent, and charring agent is anticipated. Phytic acid (PTA), with twelve negatively charged phosphoric acid groups, serves as an effective acid source. It also readily interacts with cationic materials to form aggregates [19]. Piperazine (PA) is a heterocyclic compound containing two nitrogen atoms, exhibiting notable functions as both a blowing agent and a charring agent [20]. Self-assembly technology utilizes non-covalent interactions between molecules (such as hydrogen bonds, π–π interactions, and electrostatic forces) to construct aggregates with special properties [19]. PTA and PA carry negative and positive charges in aqueous solutions, respectively, allowing them to readily self-assemble into a three-in-one IFR through electrostatic interactions.

In this work, we demonstrate a facile approach to constructing dual-modified hybrid aerogel, which is assembled by organic CS, inorganic SA, and phytate-piperazine (PPA). The CS provides a biocompatible and porous framework. SA, with its inorganic nature, can enhance the thermal stability and mechanical strength of the aerogel. PPA acts as a cross-linker and flame retardant, forming strong interactions with both CS and SA to improve the overall performance. The effect of the mass ratio of PPA to CS cross-linked to SA (CA) on the physical, flame retardancy, and mechanical properties of CA/PPA aerogels was explored. This work introduces a feasible strategy to produce high-performance hybrid aerogels with promising applications in construction, aerospace, and thermal insulation.

## 2. Experiment

### 2.1. Materials

Chitosan (CS, degree of deacetylation ≥ 95%, viscosity: 100–200 mPa·s, Mw: 22 kDa), phytic acid (PTA, 70% in water), piperazine (PA, AR), glacial acetic acid (AR), and ethanol (AR) were purchased from Aladdin Reagent Co., Ltd. (Shanghai, China). Tetraethyl orthosilicate (TEOS, AR), hydrochloric acid (1.0 mol/L), and ammonia solution (0.3 mol/L) were obtained from Shanghai Macklin Biochemical Technology Co., Ltd. (Shanghai, China). All reagents were used as received without further purification.

### 2.2. Preparation of PPA and CA/PPA Composite Aerogel

#### 2.2.1. Designing of CA/PPA Composite Aerogel

**Figure 1:**
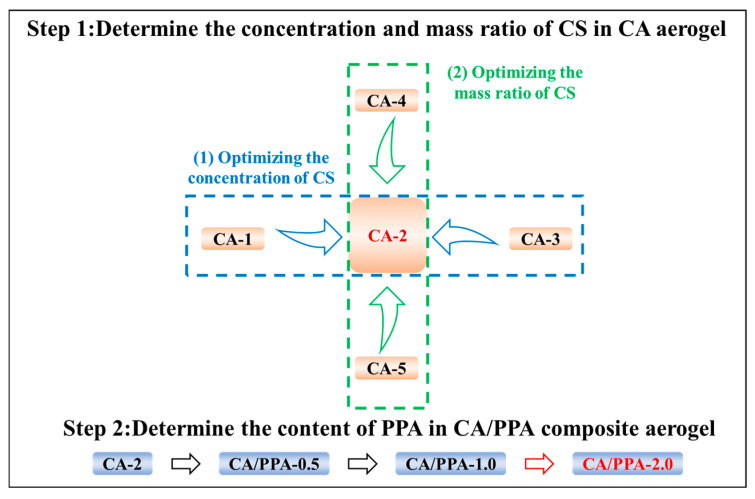
The research framework of this study.

The concentration of CS solution and the content of flame retardants substantially impact the performance of the target aerogel [21]. The composition ratios of the CA/PPA composite aerogels are listed in Table 1. Figure 1 depicts the study framework for this study. Briefly, the samples of CA-1, CA-2, and CA-3 are used to determine the optimized concentration of CS precursor in CA composite aerogel. And, at the similar concentration of CS precursor, the CA-2, CA-4, and CA-5 were used to determine the optimized mass ratio between CS precursor and SA precursor in the composite aerogel (CA). Finally, three contents of PPA (0.5 wt%, 1.0 wt%, and 2.0 wt%) in above optimized CA composite aerogel, which are named CA/PPA-0.5, CA/PPA-1.0, and CA/PPA-2.0, respectively, were prepared.

#### 2.2.2. Designing of Intumescent Flame Retardant (PPA)

**Figure 2:**
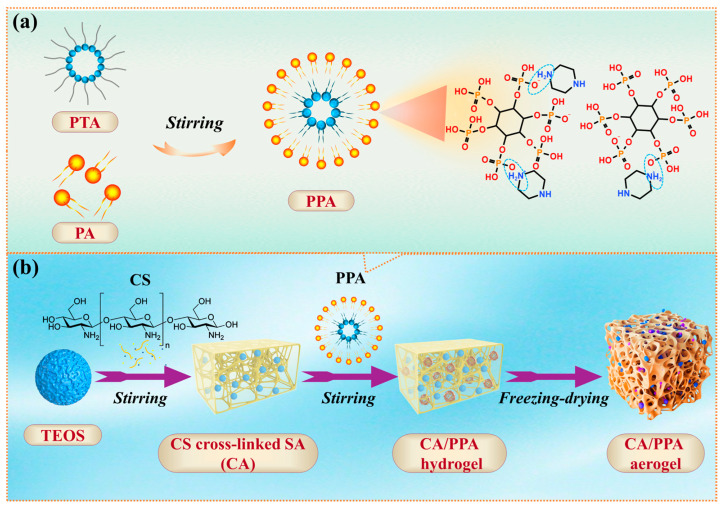
The fabrication process of (**a**) PPA and (**b**) CA/PPA hybrid composite aerogel.

The PPA was synthesized via a self-assembly technique using PTA and PA as raw materials, as shown in Figure 2a. A total of 117.5 g of PTA was first dissolved in 400 mL deionized water. A total of 45.0 g PA and 300 mL deionized water were added to a three-necked flask, followed by 20 min of stirring at room temperature for homogeneous dispersion. After the above PA solution was heated to 80 °C, the PTA solution was slowly dripped with stirring. This reaction between PTA and PA was continued for 30 min. After that, the resultant precipitate was centrifuged and subjected to three cycles of washing with 600 mL deionized water. Finally, the precipitate (phytate-piperazine, PPA) was dried at 80 °C to remove water.

The CA/PPA composite aerogel was fabricated using a self-assembly technique, followed by freeze-drying, utilizing CS, TEOS, and PPA as raw materials, as illustrated in Figure 2b. For instance, the CA/PPA-1.0 aerogel (with a CS precursor concentration of 2.0 g/100 mL, a mass ratio of 6:1 between the CS precursor and SA, and a PPA content of 1.0 wt% in the CA/PPA system) serves as an example, and the routes are outlined as follows:

Take CA/PPA-1.0 aerogel (the concentration of CS precursor is 2.0 g/100 mL; the mass ratio between CS precursor and SA is 6:1; PPA content in CA/PPA system is 1.0 wt%) as an example, and the routes are presented as follows. A total of 8 g of CS was added to 400 mL acetic acid aqueous solution (the mass ratio of acetic acid and deionized water is 4:96) and magnetically stirred at 60 °C for 5 h to form a homogeneous CS precursor solution. A mixture of TEOS, ethanol, and deionized water (volume ratio 3:6:1) was magnetically stirred for 30 min. Hydrochloric acid was added to adjust the pH to 2–3. The above solution was then heated at 60 °C with stirring for 2.5 h to prepare the SA precursor solution. The SA precursor and CS precursor solutions (mass ratio 1:6, total mass is 99.0 g) were blended by magnetic stirring for 5 min and ultrasonicated for 30 min to ensure uniform dispersion. A total of 1.0 g of PPA powder was then added to the 99.0 g mixed precursor solution. After the above aqueous dispersion was completely solidified in a −35 °C freezer, it was lyophilized in a freeze-dryer (−65 °C, 0.1 Pa) for 72 h. Finally, the CA/PPA-1.0 composite aerogel was obtained.

### 2.3. Characterization

Fourier transform infrared (FTIR) spectrum was obtained from a Nicolet IS5 spectrometer (Thermo Fisher Scientific Inc.). (Shanghai, China).

Hydrogen spectroscopy nuclear magnetic resonance (^1^H-NMR, Bruker Avance NEO 400 MHz type, Bruker Corporation, Karlsruhe, Germany) was used to characterize the sample structure. The solvent used in the ^1^H-NMR test was heavy water (D_2_O) (Bruker Corporation Inc.). (Beijing, China).

X-ray photoelectron spectroscopy (XPS) spectra were recorded by an ESCALAB 250Xi (Thermo Fisher Scientific Inc.). (Shanghai, China).

The thermal stability of the material was evaluated by thermogravimetric analysis (TGA) using the STA 6000 synchronous thermal analyzer (PerkinElmer Inc.). (Shanghai, China). At a rate of 20 °C/min, the samples (5.0 mg) were heated from room temperature.

Thermogravimetric analysis/Infrared spectrometry (TG–IR) was recorded on a PerkinElmer TGA analyzer (PerkinElmer Inc.), (Shanghai, China).which was interfaced to a Fourier transform infrared spectrophotometer. The samples were heated in helium atmosphere at a heating rate of 20 °C/min.

Micro-morphology of the powder and surface of the composite aerogel was observed from a scanning electron microscope (SEM, Gemini 300, Carl Zeiss Jena) (Shanghai, China). coupled with an energy dispersive X-ray spectrometer (X-MaxN 20, Oxford Instruments). (Shanghai, China). Before use, the samples were pre-treated by coating with a gold layer.

The mechanical performance of the sample was evaluated using a universal mechanical testing machine (Exceed Series 40, MTS Systems Co., Ltd.) (Shanghai, China) in accordance with the ASTM D6641 standard [22], at a compression rate of 3 mm/min.

The limiting oxygen index (LOI) value was tested on an HC-2C oxygen-index apparatus (Nanjing Jiangning Analytical Instrument Co., Ltd.) (Nanjing, China) by using an ASTM D2863 [23] with sample dimensions of 100 × 6.7 × 3 mm^3^.

A UL-94 vertical burning rating was obtained on a CZF-4 vertical testing apparatus (Nanjing Jiangning Analytical Instrument Co., Ltd.) according to ASTM D3801 [24].

The heat release performance of the CA-based composite aerogel was tested by a micro-scale combustion calorimeter test (MCC, Fire Testing Technology Ltd.) (East Gristead, West Sussex, United Kingdom) according to ASTM D7309-2007 [25]. The samples were heated at a rate of 3 °C/s from 100 to 800 °C.

The cone calorimeter test (Motis Fire Tech Technology Co., Ltd.) (Ningbo, China)was performed according to ISO 5660-3:2012 [26] with a thermal irradiation intensity of 50 kW/m^2^. The sample size was 100 × 100 × 4 mm^3^.

The thermal conductivity was obtained from a hot disk thermal analyzer (TPS 2500S, Hot Disk AB, Gothenburg, Sweden) under 25 °C (Hot Disk AB, Ltd.) (Shanghai, China).

A Raman microscope (LabRAM HR, HORIBA Scientific) (Shanghai, China) with excitation at 514 nm was employed for the Raman spectra with a range of 800–2000 cm^−1^.

## 3. Results and Discussion

### 3.1. Characterization of PPA Flame Retardant and Its Composite Aerogel

#### 3.1.1. Characterization of PPA Flame Retardant

The structure and morphology of the PPA was obtained by using Fourier transform infrared (FTIR) spectroscopy, nuclear magnetic resonance (NMR) spectrum, X-ray photoelectron spectroscopy (XPS) spectrum, thermal gravity analysis (TGA), and a scanning electronic microscope (SEM).

**Figure 3:**
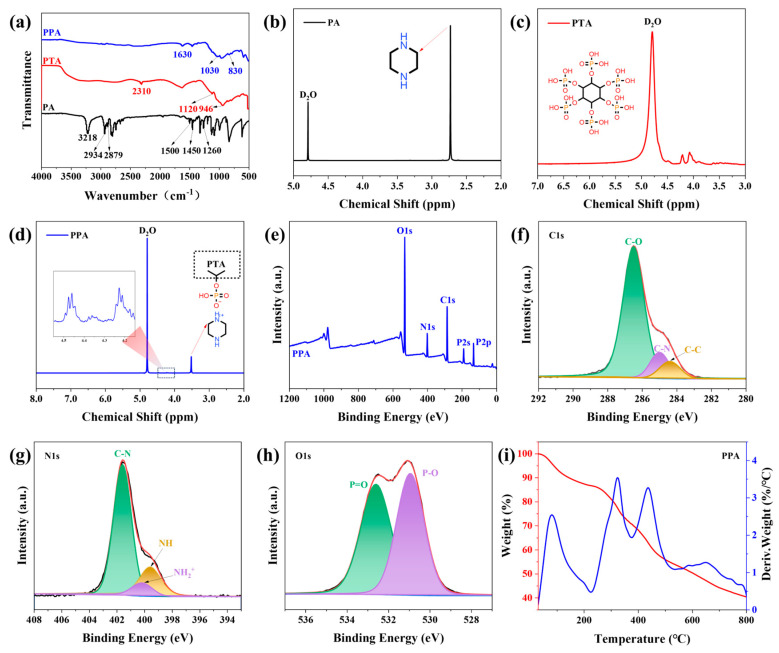
(**a**) FT-IR spectra; ^1^H-NMR spectrum of (**b**) PA, (**c**) PTA and (**d**) PPA; (**e**–**h**) XPS spectra of PPA; (**i**) TGA and DTG curves of PPA powder.

Figure 3a presents the FTIR spectroscopy of samples. For PTA, the characteristic peaks of P=O (1120 cm^−1^) and P-O (946 cm^−1^) belonged to the PO_4_^3−^ group. For PA, the peak at 3218 cm^−1^ was assigned to the asymmetric stretching vibration of N-H [27], while the peaks at 2934 and 2879 cm^−1^ were associated with CH_2_ vibrations [28]. Additionally, the peaks at 1500, 1450, and 1260 cm^−1^ were attributed to the N-H functional group, CH_2_ scissoring deformation, and C-H stretching vibrations, respectively [29]. In the FTIR spectrum of PPA, the characteristic absorption peaks of both PTA and PA were observed. The peaks at 1030 and 830 cm^−1^ corresponded to the symmetric and asymmetric stretching vibrations of P-O [30]. Notably, the C-N-H characteristic peak shifted from 1500 to 1630 cm^−1^ due to the ionic interaction between PTA and PA, indicating the formation of NH_2_^+^ [31]. Furthermore, the disappearance of the peak at 3218 cm^−1^ was attributed to the bending vibration of NH_2_ in PA [32].

^1^H NMR spectroscopy of samples is presented in Figure 3b–d. The peak at 4.8 ppm in all spectra corresponds to the solvent D_2_O. For the ^1^H NMR spectrum of PA, the peak at 2.7 ppm is attributed to the -CH_2_ protons. In the spectrum of PTA, the weak peaks at 4.1 and 4.2 ppm are associated with the -CH protons in the six-membered carbon ring [32,33], while the -OH proton signals are obscured by the solvent D_2_O [34]. In the ^1^H NMR spectrum of PPA, the weak peak near 4.0 ppm corresponds to the -CH protons of PTA after the reaction. Notably, the chemical shift in the -CH_2_ protons from PA shifts from 2.7 ppm to 3.5 ppm, which is ascribed to the formation of -CH_2_-NH_2_^+^, indicating the generation of a phytate through the reaction between PTA and PA [31,32,33].

As presented in Figure 3e–h, the XPS survey spectrum of PPA exhibited characteristic peaks for C, N, O, and P. In the high-resolution C1s XPS spectrum of PPA (Figure 3f), the peaks could be deconvoluted into three components: C-O (286.5 eV), C-N (285 eV), and C-C (284.4 eV) [19,35]. In the high-resolution N1s spectrum (Figure 3g), the peaks were assigned to C-N (401.6 eV), NH_2_^+^ (400.2 eV), and N-H (399.6 eV) [20]. For the high-resolution O1s spectrum (Figure 3h), two distinct peaks corresponding to P=O (532.61 eV) and P-O (530.95 eV) were resolved [13]. The results above indicate the successful self-assembly reaction between PTA and PA to form PPA. The detailed elemental composition of PPA, as determined from the XPS spectra, is presented in Table S1.

Figure 3i shows the TGA and DTG curves of PPA under a nitrogen atmosphere. The thermal decomposition process of PPA primarily occurs in three main stages. The first weight loss stage ranges from the initial temperature to 236.7 °C, with a weight loss of 13.5%, attributed mainly to the loss of a small amount of free water [36]. The second weight loss stage occurs between 236.7 and 504.4 °C, exhibiting a weight loss of 30.8% and the fastest weight loss rate. During this stage, cross-linked polyphosphoric acid is formed, accompanied by the release of gases such as NH_3_ and H_2_O [37]. The third weight loss stage spans from 504.4 to 800 °C. In this stage, phosphoric acid volatilizes or undergoes dehydration and decomposition, producing highly stable carbon species. The residual char yield of PPA at 800 °C is 40.5% [38].

**Figure 4:**
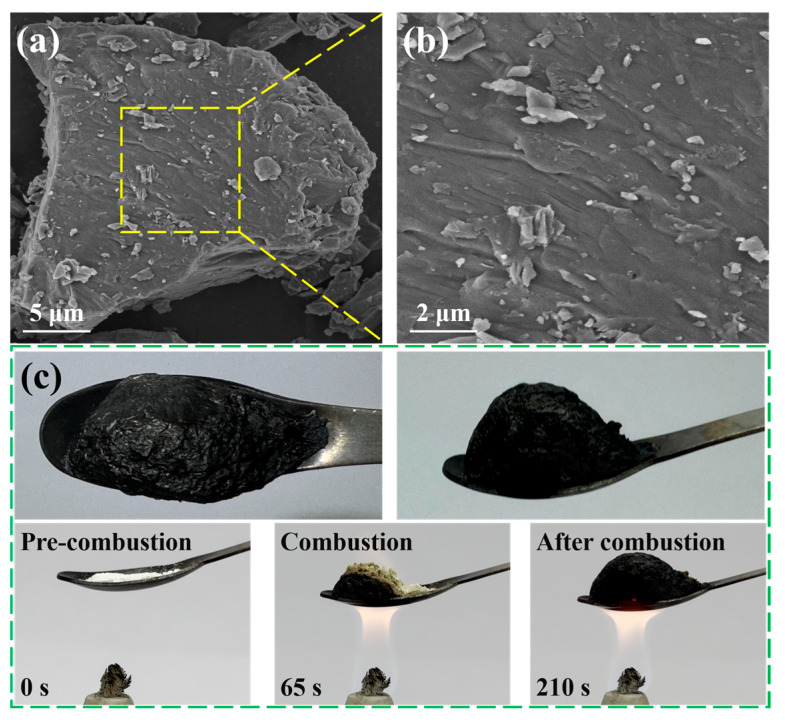
(**a**,**b**) SEM image of PPA flame retardant; (**c**) front view and top view of the expanded char layer after burning of PPA flame retardant; the IFR performance of PPA flame retardant under heating is presented.

Figure 4a,b shows the SEM image of PPA. The surface of PPA exhibits a distinct multilayered structure with roughness, which provides indirect evidence that PA and PTA undergo supramolecular assembly through cation–anion interactions to form aggregates. Figure 4c displays the IFR performance of the PPA flame retardant under heating. The PPA powder appears faint yellow prior to heat exposure. Upon ignition of the alcohol lamp, heat is transferred to the lower portion of the spoon. After it absorbs significant heat, the PPA begins to thermally release non-flammable gases, causing the char layer to expand and eventually forming a dense and stable protective charring layer [39].

#### 3.1.2. Characterization of Composite Aerogel

**Figure 5:**
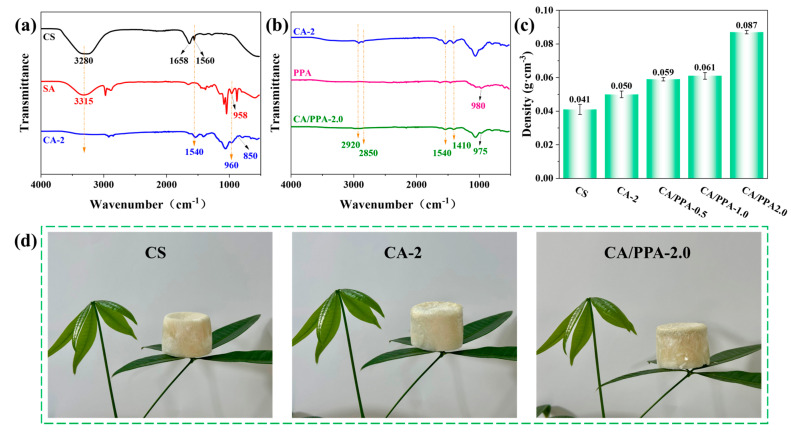
FTIR spectra of (**a**) CS, SA, and CA-2 aerogels and (**b**) CA-2, PPA and CA/PPA-2.0 aerogels; (**c**) density data plot of the CA/PPA composite; (**d**) photograph of composite aerogel under leaves.

The FTIR spectra of CS aerogel, SA aerogel, and the CA-2 aerogel are presented in Figure 5a. The FTIR spectrum of CS exhibits several characteristic bands at 3280 cm^−1^ (O-H and N-H stretching vibrations), 1658 cm^−1^ (C-N stretching vibration of amide I), and 1560 cm^−1^ (N-H bending vibration of amide II) [40]. The peaks at 958 cm^−1^ and 3315 cm^−1^ in SA correspond to Si-OH bending vibrations [4]. Notably, the peak at 3280 cm^−1^ in CS and the peak at 3315 cm^−1^ in SA are absent in the FTIR spectrum of CA-2 within the 3000–3500 cm^−1^ range [10]. This absence is attributed to the formation of ionic bonds and hydrogen bonds between the NH_2_ groups of CS and the Si-OH groups of SA. Additionally, the absorption bands at 1560 cm^−1^ in CS and 958 cm^−1^ in SA are shifted to 1540 cm^−1^ and 960 cm^−1^, respectively, in CA-2, indicating the formation of hydrogen bonds during the interaction between CS and SA [41]. Furthermore, a new peak corresponding to Si-N-C is observed at 850 cm^−1^ in CA-2 [10]. These findings suggest that the condensation reaction between SA (providing Si-OH) and CS (providing -NH_2_) facilitates the formation of a stable Si-N-C structure, with the hydrogen bond interactions between SA (providing Si–OH) and CS (providing -OH) playing a crucial role in forming cross-linked CA-2.

The FTIR spectra of CA-2 aerogel, PPA, and CA/PPA-2.0 aerogel are presented in Figure 5b. The stretching vibration peak of 2920, 2850, 1540, and 1410 cm^−1^ (typical peaks for CA-2) is also observed in CA/PPA-2.0 aerogel [21]. Furthermore, the shift in the PO^3−^ peak from 980 cm^−1^ for PPA to 975 cm^−1^ for CA/PPA-2.0 may be attributed to the inductive effect between PO^3−^ groups and NH_2_^+^ groups [42]. This result would portend abundant hydrogen bonding and/or π–π interaction occurring between PO^3−^ groups in the PPA and NH_2_^+^ groups in the crosslinked CA-2 aerogel [42,43]. That is, a dual-crosslinked CA/PPA-2.0 can be prepared by the reaction between CS, SA, and PPA.

Figure 5c presents the density of the CA/PPA composite aerogels, while Figure 5d visually illustrates the low-density characteristics of the aerogel through photographs. The density of the CS cross-linked SA composite aerogel demonstrates an increasing trend with higher PPA loading; however, all composite aerogels remain stable on the leaves.

**Figure 6:**
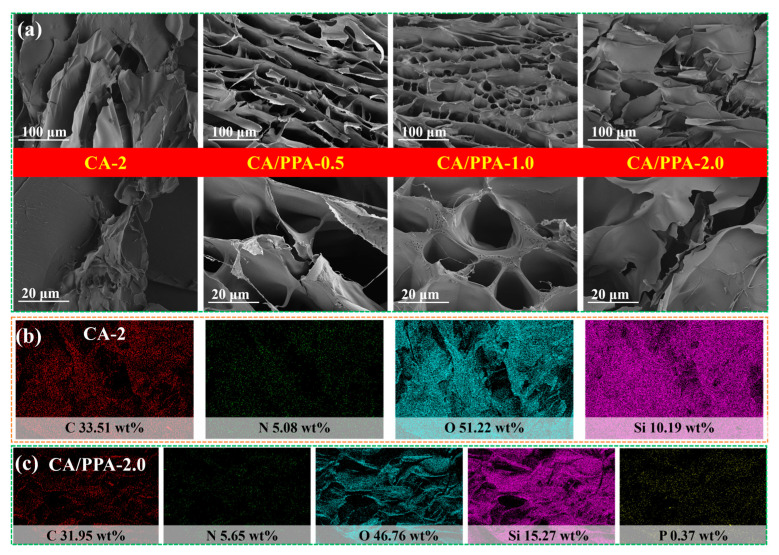
(**a**) SEM image of CA/PPA; elemental mapping images of (**b**) CA-2 and (**c**) CA/PPA-2.0.

SEM was further employed to observe the cross-section morphology of CA-2 and flame-retardant CA/PPA composite aerogels. As Figure 6a presents, all the composite aerogels exhibit a porous structure. The low thermal conductivity and thermal insulation is also ascribed to these unique 3D structures and nano/micron-pores. As with the elemental mapping images presented in Figure 6b, the typical elements of C and N belonging to CS and the typical element of Si belonging to SA are present in CA-2 aerogel, indicating a cross-linked effect between CS and SA. Furthermore, the elemental mapping image of P (Figure 6c) corroborates the homogeneous distribution of the PPA constituent in the CA/PPA-2.0 aerogel. The exceptional compatibility of PPA within the CA matrix played a crucial role in maintaining the well-layered porous architecture of the aerogel [44]. Consequently, this enhanced the thermal insulation properties exhibited by the CA/PPA-2.0 composite aerogel.

### 3.2. Optimizing the Compound of CA Based on Its Mechanical and Fire Safety Properties

A CA composite aerogel, where CS is cross-linked to SA, has its performance influenced by the concentration and content of the CS solution. For energy-efficient buildings, materials with high fire resistance and mechanical properties are essential. Therefore, this work investigates the concentration and content of the CS solution in CS cross-linked SA aerogel (CA) based on its mechanical properties. Specifically, samples CA-1, CA-2, and CA-3 are used to determine the optimal concentration. Meanwhile, at a similar concentration of the CS precursor, samples CA-2, CA-4, and CA-5 are employed to identify the best mass ratio between the CS precursor and the SA precursor. The research framework of this study is also depicted in Figure 2.

#### 3.2.1. Determine the Optimal Concentration of CS in CA

**Figure 7:**
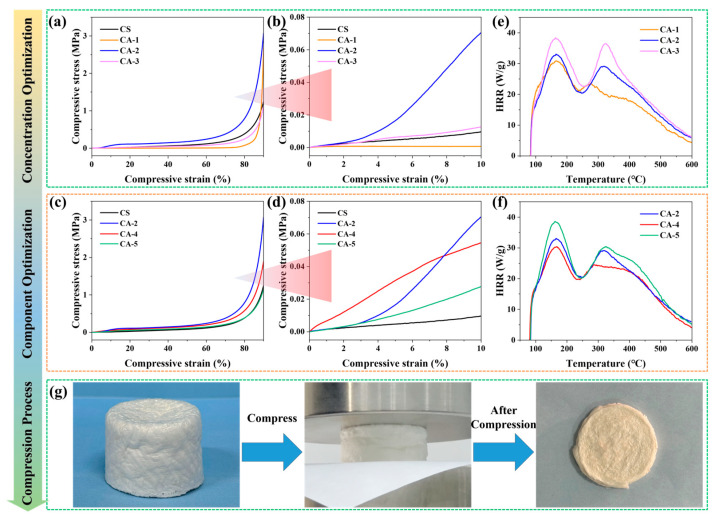
(**a**–**d**) Compressive stress–strain curves of the aerogels; (**e**,**f**) HRR curves of aerogels under MCC test; (**g**) photographs of the compression process of CA-2.

The CA composite aerogels with three concentrations of CS acetic acid aqueous solution (1.0, 2.0 and 3.0 g/100 mL) were named CA-1, CA-2, and CA-3, respectively. As with the mechanical properties presented in Figure 7a,b,g, all of these aerogels demonstrated favorable stress resistance at 90% compression, indicating their inherent toughness. Figure 7e presents the heat release rate (HRR) versus temperature curves of the samples, as obtained from micro-scale calorimeter (MCC) tests. The two components—chitosan and silica—display distinct thermal degradation behaviors, resulting in two different peak heat release rates (PHRR_m_) for the CA composite aerogel [45]. During MCC testing, the PHRR_m_, temperature at PHRR_m_ (*T*_PHRR_) and total heat release (THR_m_) were applied to evaluate the flame-retardant properties. Figure S1 also presents the compressive stress and HRR curves of CS aerogel. The data regarding the mechanical and fire safety properties of CS and its composite aerogels is also summarized in Table S3.

As shown in Figure 7a,b and Table S3, with increasing concentrations of CS in the CA, the mechanical properties of the CA composite aerogel exhibit a trend of first increasing and then decreasing. The compressive strength at 90% compression stress of the CA-2 aerogel is 3.1 ± 0.25 MPa. The optimal concentration of CS is 2.0 wt%. At this concentration, the following section continues to explore the mass ratio of CS to SA.

#### 3.2.2. Determine the Optimal Ratio of CS in CA

The CA composite aerogels with three mass ratios of CS to SA (4:1, 6:1, and 8:1) were named CA-4, CA-2, and CA-5, respectively. The compressive strength and HRR curves of these composite aerogels are presented in Figure 7c and d and Figure 7f, respectively. The data regarding the mechanical and fire safety properties of CS and its composite aerogels is also summarized in Table S3. As is shown, with an increasing loading of CS in the CA, the mechanical properties of the CA composite aerogel exhibit a trend of first increasing and then decreasing. The compressive strength at 90% compression stress of the CA-2 aerogel is 3.1 ± 0.25 MPa. Therefore, the optimal mass ratio of CS to SA in the CA is determined to be 6:1.

Based on the above analysis, the optimized concentration of CS solution and the mass ratio of CS to SA in CA composite aerogel are set as 2.0 g/100 mL acetic acid aqueous solution and 6:1, respectively. Figure 7e,f and Figure S1 and Table 2 exhibit the fire-retardant behavior of CA composite aerogels. Lower heat release capacity (HRC; HRC = PHRR_m_/heating rate), PHRR_m_, and THR_m_ indicate greater fire safety [14,19,46]. Unfortunately, as the concentration and content of CS increase, the fire risk of its composite aerogel also rises accordingly. For energy-efficient buildings, materials with high fire resistance and mechanical properties are essential. Therefore, at this CA-2 composition, the subsequent text investigates the mass loading of PPA (a novel IFR) to further develop high-performance (high mechanical and fire safety properties), dual-modified hybrid aerogels.

### 3.3. Fire Behaviors of PPA-Based CA

#### 3.3.1. Flame Retardancy Performance of PPA-Based CA

The flame retardancy performance was assessed through various methods including limiting oxygen index (LOI), UL-94 vertical burning, MCC, and cone calorimeter (CONE) apparatus. The related data collected from MCC (peak heat release rate: PHRR_m_, temperature at PHRR_m_: *T*_PHRR_) and CONE (time to ignition: *t*_ign_, peak heat release rate: PHRR_c_, time at PHRR_c_: *t*_PHRR_, total heat release: THR_c_) is presented in Table 2.

**Figure 8:**
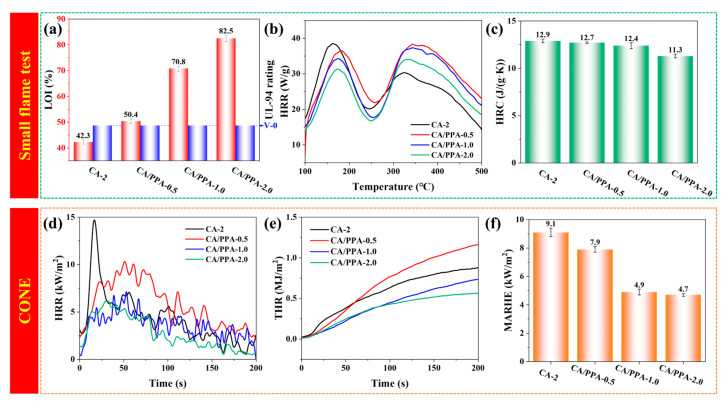
(**a**) LOI value and UL-94 test data; (**b**) HRR and (**c**) HRC from MCC tests; (**d**) HRR as a function of time from CONE, (**e**) THR and (**f**) MARHE from CONE.

As presented in Figure 8a, with loading 0.5, 1.0, and 2.0 wt% PPA in CA-2, their LOI values increased from 42.3% to 50.4%, 70.8%, and 82.5%, respectively. In the UL-94 test, all CA/PPA composite aerogels achieved a V-0 rating with no melt dripping. The HRR versus temperature curves and the HRC of the aerogels, obtained from MCC tests, are presented in Figure 8b,c. The two components—chitosan and silica—display distinct thermal degradation behaviors, resulting in two different PHRR_m_ for the CA composite aerogel [45]. With loading 0.5, 1.0, and 2.0 wt% PPA in CA-2, their HRC values decreased from 12.9 J/(g·K) to 12.7, 12.4, and 11.3 J/(g·K), respectively. CONE provides essential information regarding the flammability and smoke emission of composites, with the associated data correlating well with full-scale fire tests [20]. The HRR and THR versus time curves, and the maximum average rate of heat emission (MARHE) of the aerogels, obtained from CONE tests, are shown in Figure 8d–f. The CA/PPA composite aerogels with three contents of PPA (0.5, 1.0, and 2.0 wt%) were named CA/PPA-0.5, CA/PPA-1.0, and CA/PPA-2.0, respectively. CA-2 ignites rapidly within 12 s. However, all CA/PPA composite aerogels remain in a smoldering state with no obvious flame. As presented in Figure 8, with an increasing loading of PPA, the values of PHRR_m_, PHRR_c_, and THR_c_ of their composite aerogel exhibit a trend of decreasing. Compared to CA-2, the values of PHRR_m_, PHRR_c_, and THR_c_ of CA/PPA-2.0 decreased by 18.9%, 62.1%, and 55.6%, respectively.

The derived data regarding the fire growth index (FGI = PHRR_c_/*t*_PHRR_) is listed in Table 2. Enhanced fire safety is associated with lower FGI, MARHE (Figure 8f), and HRC (Figure 8c) values. Among all the composite aerogels, CA/PPA-2.0 obtained the lowest values of FGI (0.15 kW/m^2^s). MARHE (4.7 kW/m^2^) and HRC (11.3 (J/g·K)) indicated high fire safety properties [14,19,46].

To further highlight the superior flame-retardant efficiency of CA/PPA-2.0, the fire behavior property of CS-based aerogel composites is compared to previous works, as shown in Figure S2. Additionally, Table S4 lists the detailed data and corresponding references. It is noted that the flame-retardant efficiency of CA/PPA-2.0 is also effective, as compared with the CS-based aerogel composites, such as **TCS-5.0** (Phosphorus-containing aldehyde/Chitosan aerogels) [47], **CCA 2** (Chitosan Glutaraldehyde aerogels) [48], **CA1.5P-AIP1** (Chitosan-aluminum/PVA (CAP) aerogels and aluminum isopropoxide (AIP)) [49], **PCS-6** (Phosphorylated chitosan) [50], **CSA-HGM-Mg(OH)_2_** (Incorporation of Mg(OH)_2_ coated hollow glass microspheres (HGM) into chitosan (CSA) matrix and then cross-linking by glutaraldehyde) [51], and **LBL6** (Cellulose filaments/phytic acid/chitosan aerogels) [52].

#### 3.3.2. Thermal Insulation and Fire Resistance Properties of PPA-Based CA

**Figure 9:**
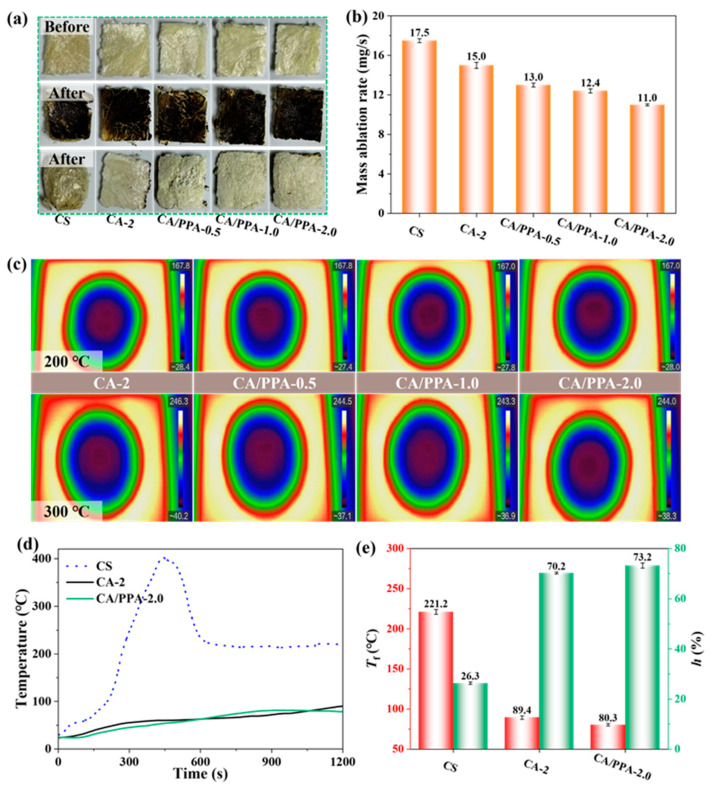
(**a**) Visible fire resistance effects of aerogels and (**b**) their mass ablation rate; (**c**) thermal images of composite aerogels; (**d**) the temperature curves of aerogels during heating until thermal equilibrium and (**e**) their corresponding data (values of *h* and *T*_f_).

The fire resistance evaluation of the aerogels was conducted under 10 s sustained ignition conditions, as detailed in Figure S3. The continuous fire source was a flame gun fueled by acetylene gas, with a flame temperature capable of reaching 1000 °C. The test results in Figure 9a reveal that neat CS aerogel burned quickly. Furthermore, all CS, CA-2, and CA/PPA-0.5 aerogels exhibited significant shrinkage when exposed to fire. Cross-sectional images of CA/PPA-1.0 and CA/PPA-2.0 aerogels reveal that the interior retained its original morphology and color after combustion, demonstrating that PPA effectively protected the inner CA-2 matrix. It is notable that increasing the PPA loading in CA-2 resulted in a more complete carbonized layer on the surface. Specifically, the CA/PPA-2.0 aerogel formed a continuous carbon layer, which prevented the transfer of flames and heat. The mass ablation rate (the change in sample mass before and after the test divided by the test time) is applied to evaluate the fire resistance, and presented in Figure 9b. A lower mass ablation rate indicated a higher fire resistance [11]. Among all of the aerogels, the CA/PPA-2.0 aerogel obtained the lowest mass ablation rate, indicating the excellent fire resistance.

The thermal infrared imaging experimental setup is illustrated in Figure S4 [11,53]. Test samples were placed on a temperature-adjustable heating stage set to 200 and 300 °C. The surface temperatures at the top of these samples were recorded using the data processing software of the infrared thermal imaging camera. As shown in Figure 9c, all composite aerogels’ surfaces exhibited neither cracks nor contractions, demonstrating their favorable thermal-isolating performance. Upon the thermal degradation of PPA, PTA releases phosphorus-rich acids that react with PA at elevated temperatures, promoting the intermolecular cross-linking of polyphosphoric backbones. This reaction increases the fraction of the CA/PPA aerogel available for phosphorylation and subsequent char formation. Consequently, PPA functionalization, together with the inherent physical barrier characteristics of the CA/PPA network, collectively enhance the thermal insulation and fire-retardant performance of the composite aerogel [11,14,19,53]. The thermal conductivity of the CS aerogel, CA-2 aerogel, and CA/PPA-2.0 aerogel was 0.048 W/m·K, 0.039 W/m·K, and 0.042 W/m·K (Table S2), respectively. Although the thermal conductivity of the CA/PPA-2.0 aerogel was slightly higher than that of the CA-2 aerogel, it still satisfied the performance requirements for insulation boards (≤0.080 W/m·K, GB50264-2013) [54].

An improved laboratory setup, based on the large plate method [11], was employed to further investigate the thermal insulation performance of the aerogel, as illustrated in Figure S5. Figure 9d exhibits the temperature curves of composite aerogels during heating until thermal equilibrium is reached. As shown, with continued fire heating, the surface temperature of the neat CS aerogel exhibits a trend of initially increasing and then decreasing. The initial increasing stage corresponds to the combustion behavior of the CS aerogel, which is corroborated by MCC and CONE tests (Figure S1). The subsequent decreasing stage is attributed to the formation of its combustion-induced carbonized structure [53]. Compared to neat CS aerogel, both CA-2 and CA/PPA-2.0 composite aerogels exhibit super thermal insulation performance. The corresponding data, including the insulation degree (*h*, calculated according to Equation (1)) and final temperature (*T_f_*) of the composite aerogel, is presented in Figure 9e. Higher *h* and lower *T_f_* values correspond to the enhanced thermal insulation property of aerogels [11]. The exceptional compatibility of PPA within the CA matrix played a crucial role in maintaining the well-layered porous architecture of the aerogel. Consequently, this enhanced the thermal insulation properties exhibited by the CA/PPA-2.0 composite aerogel. Among all tested samples, CA/PPA-2 achieved the highest *h* (73.2%) and lowest *T_f_* (80.3 °C) value, indicating its excellent thermal insulation properties and the exceptional compatibility of PPA within the CA matrix.

(1) $$h=\frac{T_{N}-T_{f}}{T_{N}}$$

where *T_N_* and *T_f_* are the final surface temperatures at the back of the steel and aerogel, respectively. *T_N_* was tested as 300 °C.

Based on the above analysis, the CA/PPA-2.0 aerogel displayed the highest thermal insulation and fire resistance among all samples. Thus, incorporating 2.0 wt% PPA into CA-2 represents an efficient strategy to enhance the thermal insulation and fire resistance performance of the composite aerogel.

#### 3.3.3. Thermal Stability of PPA-Based CA

Figure S6a–d displays the TGA and DTG curves of the composite aerogels. The maximum mass loss rate (MLR_max_), char residue at 800 °C (CR), temperatures at 5% mass loss (*T*_5%_), and the temperature at MLR_max_ (*T*_d_) are summarized in Table S5. For PPA-containing composite aerogels, *T*_5%_ and *T*_d_ shift to lower temperatures and MLR_max_ increases, owing to early decomposition of the intumescent flame retardant or its catalytic effect on the base aerogel [55]. The CR of CA/PPA-2.0 (54.5%) is higher than that of CA-2 (40.2%). This is ascribed to the production of thermally stable char from the charring reactions between PPA.

### 3.4. Flame Retardancy Mechanisms

The flame retardancy mechanisms of PPA-based CA is proposed based on the analysis of the gaseous phase and condensed phase product.

#### 3.4.1. Analysis of Gaseous Phase Product

**Figure 10:**
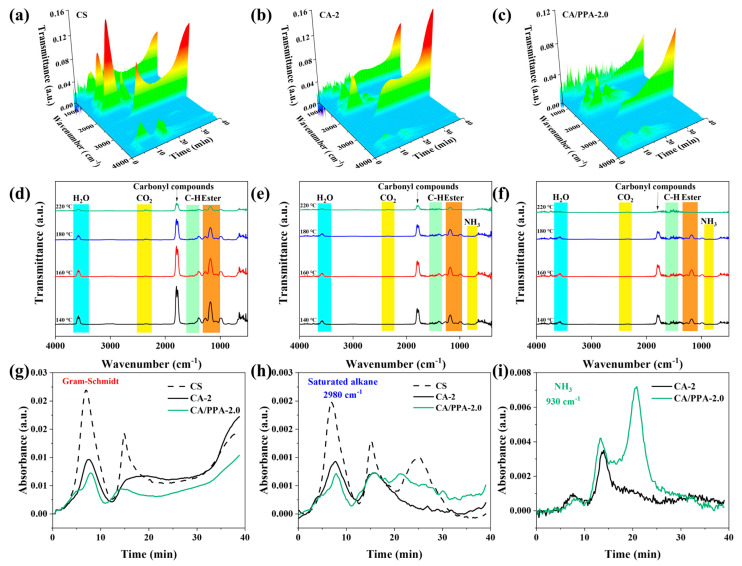
(**a**–**c**) 3D TG-IR spectrum; FTIR spectra of (**d**) CS, (**e**) CA-2, and (**f**) CA/PPA-2.0 at different temperatures; (**g**) Gram–Schmidt curves; absorbance of (**h**) saturated alkanes and (**i**) NH_3_.

The pyrolysis gases of pure CS, CA-2, and CA/PPA-2.0 were investigated using thermogravimetric analysis-infrared spectroscopy (TG-IR). During gaseous product evolution, all samples exhibited comparable characteristic peaks within identical temperature ranges (Figure 10a–f). The detected pyrolytic volatiles primarily comprised H_2_O (3580–3450 cm^−1^), hydrocarbons (1414 and 1372 cm^−1^), CO_2_ (2361 and 2323 cm^−1^), carbonyl compounds (1762 cm^−1^), and esters (1250–1010 cm^−1^) [11,17,20,56,57]. At equivalent temperatures (Figure 10d–f), differential peak intensities emerged at identical characteristic bands. At 160 °C, CA/PPA-2.0 exhibited stronger intensities for H_2_O, hydrocarbons, and esters in its gaseous products compared to reference samples. Conversely, its CO_2_ intensity was attenuated. Significantly reduced peak intensities for CA/PPA-2.0 relative to CS and CA-2 were observed at 180 °C and 220 °C, indicating that PPA incorporation effectively suppresses volatile component generation through catalyzed char formation.

Three peaks in the Gram–Schmidt curves of the aerogels are exhibited in Figure 10g. Primary gaseous species released from CS, CA-2, and CA/PPA-2.0 included saturated alkanes (Figure 10h) and NH_3_ (Figure 10i). NH_3_ generation in CA-2 arises from reactions involving the nitrogen atoms intrinsic to chitosan, whereas the NH_3_ signal in CA/PPA-2.0 results from the thermal decomposition of both PPA and chitosan. CA/PPA-2.0 exhibited higher NH_3_ signal intensity than CA-2, while displaying the lowest saturated alkane absorbance among all samples. These results provide compelling evidence that PPA incorporation markedly suppresses organic volatile release while promoting the evolution of non-flammable gases (NH_3_, H_2_O, etc.). Consequently, PPA markedly enhances the flame-retardant performance of the CA [11,56].

#### 3.4.2. Analysis of Condensed Phase Product

**Figure 11:**
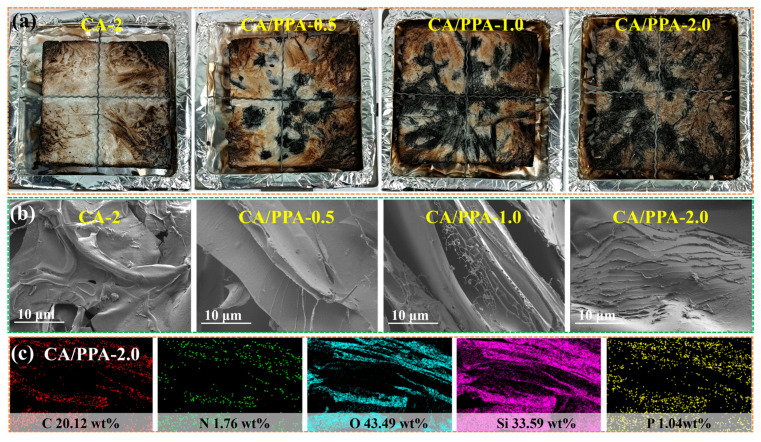
(**a**) Digital pictures of char residues of aerogels after CONE; (**b**) SEM image of the CA/PPA char residues; (**c**) EDS elemental mapping of char residues of CA/PPA-2.0.

Digital pictures of the aerogels’ char residues after CONE are presented in Figure 11a. Increasing the PPA content yields increasingly continuous char layers on the aerogel surface. To elucidate the heat- and smoke-suppression mechanisms of the aerogel, the char residue after combustion was analyzed using SEM (Figure 11b). The CA-2 yielded a relatively smooth char layer riddled with voids and fissures, which provided only limited resistance to heat and oxygen ingress. In contrast, chars from CA/PPA-0.5 and CA/PPA-1.0 displayed rougher surfaces but still contained visible cracks. The CA/PPA-2.0 sample formed a dense, continuous carbonaceous layer marked by folds and embedded white particulates; this morphology acts as an effective barrier that suppresses combustion and toxic-smoke release. Elemental mapping of the CA/PPA-2.0 char confirms a continuous network with uniformly distributed SiO_2_ throughout the aerogel (Figure 11c).

**Figure 12:**
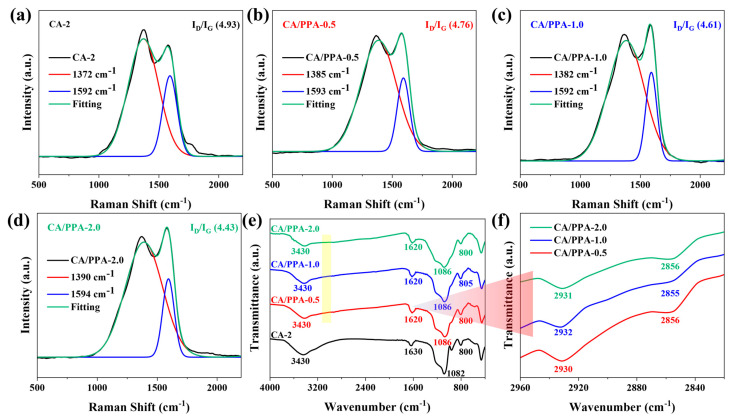
Raman spectrum of char residual of (**a**) CA-2, (**b**) CA/PPA-0.5, (**c**) CA/PPA-1.0, and (**d**) CA/PPA-2.0; (**e**,**f**) FTIR spectra of the char residual.

The residual char structure was further examined by using Raman spectroscopy. The D band (approximately 1380 cm^−1^) corresponds to disordered carbon, whereas the G band (approximately 1590 cm^−1^) arises from sp^2^-hybridized carbon. The intensity ratio (I_D_/I_G_) serves as an indicator of the graphitization degree in the residual char. A lower I_D_/I_G_ ratio signifies higher graphitization and superior char quality, which more effectively blocks the transfer of flammable gases, oxygen, and energy between gaseous and condensed phases [58]. According to Figure 12a–d, the I_D_/I_G_ ratio of CAPPA-0.5 is lower than that of neat CA-2. Specifically, CA/PPA-0.5 exhibits a value of 4.76, which declines further as the PPA content rises. This data indicates that PPA promotes the formation of a more graphitic carbon layer during combustion.

FTIR spectroscopy was employed to determine the chemical structures of the char residue after the CONE test. As presented in Figure 12e, the chemical structures of all tested samples were almost identical, mainly including O-H stretching vibration (3430 cm^−1^), C=O stretching (1630 cm^−1^), polyaromatic carbon structures (1620 cm^−1^), and O-Si-O bending (800 and 805 cm^−1^) [11,20,56,59]. In contrast to the CA-2 residue, FTIR spectra of the CA/PPA char still display C-H stretching bands (2930–2932, 2855, and 2856 cm^−1^), reflecting the elevated thermal stability conferred by the PPA [11,20,56]. It has been widely reported that the P-O-C structure endows the carbon layer with the advantages of high-temperature density, an antioxidant barrier, and structural reinforcement, significantly enhancing the flame retardancy and thermal protection performance [11,20,56]. Notably, the FTIR spectrum of the CA/PPA char (Figure 12f) shows a distinct band at 1086 cm^−1^, which is attributed to P-O-C linkages formed during PPA decomposition.

#### 3.4.3. In-Depth Flame-Retardant Mechanisms of PPA

**Figure 13:**
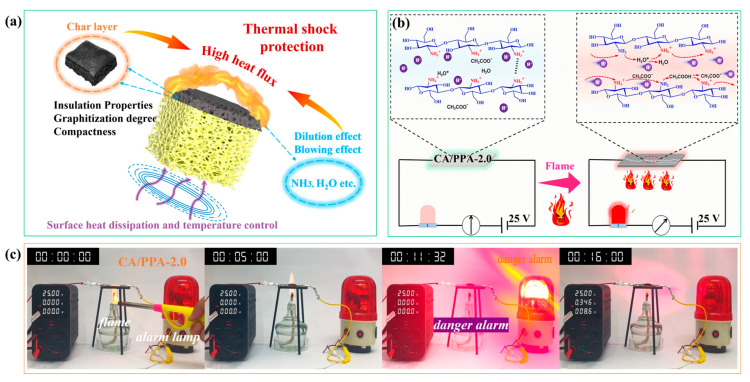
(**a**) Schematic illustrations of flame retardancy mechanism for CA/PPA composite aerogel; (**b**) fire-warning mechanism and (**c**) pictures of flame detection processes of CA/PPA-2.0.

Based on the above characterization and analysis, the flame retardancy mechanism of PPA in the CA matrix is proposed (Figure 13a). Energy-efficient buildings demand materials that combine low thermal conductivity with high fire resistance [60,61,62]. PPA first extracts substantial heat from the CA surface. Upon decomposition, it releases non-flammable gases (NH_3_ and H_2_O) that dilute oxygen and suppress the flame [28], while NH_3_ simultaneously scavenges reactive radicals in the gas phase [63,64]. During pyrolysis, phosphorus-containing species generated by PPA promote the formation of a protective char layer and produce PO• and HPO• radicals that quench H• and OH•, interrupting the combustion chain reaction [63,64]. The absorbed heat also drives PPA toward phosphoric and polyphosphoric acids, which esterify the intrinsic PA component, releasing additional NH_3_ and H_2_O and causing the melt to foam and intumesce. Upon completion, the system solidifies into a dense, intumescent char layer [28,65] that blocks mass and energy transfer, thereby inhibiting the pyrolysis of the underlying CA matrix [19,66].

### 3.5. Fire-Warning Performance and Mechanical Properties of the Composite Aerogel

Fire accidents pose significant safety risks. Developing early fire detection devices for emergency warning is an effective strategy for preventing fire-related losses [67]. The fire alarm response was investigated using a simple testing circuit composed of a low-voltage battery, an alarm lamp, wires, and a CA/PPA-2.0 aerogel, as illustrated in Figure 13b. As shown in Figure 13c, the lamp illuminated within 11.32 s of flame exposure, with a peak response current reaching 0.346 A. This rapid and sustained response underscores the composite’s potential as an effective early fire detection material, providing critical time for evacuation. Figure 13b also illustrates the fire alarm mechanism of the CA/PPA-2.0 aerogel. Upon exposure to fire, the CA/PPA-2.0 undergoes thermal decomposition, forming a conductive char layer. These results are corroborated by the SEM images in Figure 11 and the XPS spectra in Figure 12. Additionally, under fire conditions, active protons are transferred by hopping between adjacent water molecules and acetic acid ions under voltage drive, thereby forming proton conduction channels within the CA/PPA-2.0 aerogel. Moreover, the incorporation of PPA increases the number of charge carriers (PO_3_^−^ and NH_4_^+^), leading to enhanced direct current conductivity. Furthermore, the electrostatic repulsion and hydrogen bonding between CS chains are weakened, resulting in a slight reduction in the crystallinity of the composite due to the electrostatic shielding effect of PPA, which facilitates ion migration. The synergistic interaction between the CA matrix and PPA enhances the formation of a protective char layer and preserves internal electrical conductivity pathways. This results in a sharp decrease in electrical resistance, triggering an alarm signal (e.g., lighting a bulb).

The physical and mechanical properties of composite aerogels were conducted through the examination of compression strength. As seen in Figure S7a–c, with the addition of silica, the compressive strength of CA-2 aerogel was slightly improved, probably due to the nano-enhancing effect of SA [68]. With the increase in the additional amount of PPA in CA-2, the mechanical properties of its composite aerogel show a trend of first decreasing and then increasing, but all are less than those of pure CA-2 aerogel. At the stage of low PPA addition amount (0.5 wt%), interface defects were dominant [69,70]. The compressive strength of its aerogel (CA/PPA-0.5) at 90% compression decreased from 3.1 ± 0.25 MPa to 1.5 ± 0.25 MPa compared with pure CA-2. PPA particles can block the aerogel’s nanochannels, disrupt its continuous three-dimensional network, reduce the matrix’s effective load-bearing area, and thereby diminish its mechanical performance [69,70]. This is also confirmed by the morphology of fragmentation for CA/PPA-0.5 after the compression test in Figure S7d. The compressive strengths of CA/PPA-1.0 and CA/PPA-2.0 at 90% compression are markedly higher than that of CA/PPA-0.5 at the stage of high PPA addition amount (1.0 wt % and 2.0 wt%), PPA particle enhancement, and network reconstruction [69,70]. When the concentration of PPA is high enough, physical accumulation or secondary networks may form between particles (such as the skeleton effect of PPA-reinforced CA), sharing part of the external force and restoring the strength [69,70]. Although the CA/PPA-2.0 aerogel reconstructs a cross-linked network at high PPA loading, its compressive strength (2.9 ± 0.23 MPa) still falls slightly below that of pristine CA-2 aerogel (3.1 ± 0.25 MPa). The incorporation of 2.0 wt% PPA raises the density to 0.87 g cm^−3^; because aerogel mechanics are acutely sensitive to porosity (>90%), the reinforcement provided by rigid PPA particles cannot fully offset the detrimental effect of reduced porosity. To further highlight the superior mechanical efficiency properties of CA/PPA-2.0, the mechanical properties of SA-based aerogel composites are compared to previous works. Additionally, Table S6 lists the detailed data and corresponding references. It is noted that the mechanical efficiency properties of CA/PPA-2.0 are also effective, as compared with the SA-based aerogel composites, such as **WPCC/SA** (porous cellulose/silica aerogel) [71], **SiC-SiO_x_** (the laminated SiC-SiO_x_ nanowire aerogel) [72], and **Si-BN** (boron nitride/silica aerogel) [73].

## 4. Conclusions

In this work, dual-crosslinked CA/PPA aerogels were fabricated via lyophilization. The as-synthesized CA/PPA-2.0 exhibits superior mechanical properties, with its compressive strength increasing from 1.3 ± 0.10 MPa to 2.9 ± 0.23 MPa at 90% strain relative to the neat chitosan aerogel. Moreover, relative to the pristine CA, the CA/PPA composite containing 2.0 wt% PPA demonstrates markedly improved fire-safety performance. Specifically, the peak heat release rate, total heat release, and maximum average rate of heat emission were reduced by 62.1%, 55.6%, and 48.4%, respectively. This work introduces a feasible strategy for high-performance hybrid aerogels with promising applications in construction, aerospace, and thermal insulation.

## Figures and Tables

**Table 1:** Formulation of CA and CA/PPA hybrid composites.

Sample	Composition (wt%)			CS Concentration ^a^ (g/100 mL)
	SA Solution	CS Solution	PPA	
CS	-	100	-	2.0
CA-1	14.3	85.7	-	1.0
CA-2	14.3	85.7	-	2.0
CA-3	14.3	85.7	-	3.0
CA-4	20.0	80.0	-	2.0
CA-5	11.1	88.9	-	2.0
CA/PPA-0.5	14.2	85.3	0.5	2.0
CA/PPA-1.0	14.1	84.9	1.0	2.0
CA/PPA-2.0	14.0	84.0	2.0	2.0

^a^ CS concentration: the concentration of CS in 4.0 wt% acetic acid aqueous solution.

**Table 2:** MCC and CONE test data for neat CS and CA/PPA composites.

Sample	MCC		CCT				
	PHRR_m_ (W/g)	*T*_PHRR_ (°C)	*t*_ign_ (s)	PHRR_c_ (kW/m^2)^	*t*_PHRR_ (s)	THR_c_ (MJ/m^2^)	FGI (kW/m^2^s)
CS	234.3 ± 0.6	317.0 ± 0.6	3	146.2 ± 0.5	14 ± 1	3.3 ± 0.3	10.44
CA-2	38.7 ± 0.1	161.9 ± 0.2	12	15.3 ± 0.4	17 ± 1	1.8 ± 0.2	0.90
CA/PPA-0.5	36.4 ± 0.2	184.9 ± 0.3	SF ^a^	10.5 ± 0.3	51 ± 1	1.6 ± 0.1	0.21
CA/PPA-1.0	34.3 ± 0.1	174.7 ± 0.3	SF	7.6 ± 0.6	49 ± 2	1.1 ± 0.1	0.16
CA/PPA-2.0	31.4 ± 0.1	172.9 ± 0.5	SF	5.8 ± 0.3	40 ± 1	0.8 ± 0.1	0.15

^a^ SF: smoldering fire.

## Data Availability

The original contributions presented in this study are included in the article and Supplementary Materials. Further enquiries can be directed to the corresponding authors.

## Supplementary Materials

The following supporting information can be downloaded at: https://www.mdpi.com/article/10.3390/polym17233162/s1, Table S1: The detailed elemental composition of PPA obtained from XPS spectra; Table S2: Specific surface area (SBET), pore diameter (d), and pore volume (Vpore) of aerogels; Table S3: The mechanical property and MCC test data for the CA composite aerogel; Table S4: Flame retardancy results of CS-based aerogel composites in the literature and this work; Table S5: TGA data of the CA/PPA composite aerogels; Table S6: Mechanical properties of SA-based aerogel composites in the literature and this work; Figure S1: (a) HRR curves of neat CS cryogel under MCC test; (b) HRR and (c) THR curves of neat CS cryogel under CONE; Figure S2: Comparison of flame retardancy for CS-based aerogel composites; Figure S3: Schematic diagram of fire resistance evaluation experimental setup; Figure S4: Schematic diagram of thermal infrared imaging experimental setup; Figure S5: Schematic diagram of the alcohol burner experimental setup; Figure S6: (a) and (b) TGA curves, and (c) and (d) DTG curves of the composite aerogels; Figure S7: (a) and (b) Compressive stress curves of the CA/PPA composite and their (c) compressive strength; (d) Photographs of the CA/PPA composite before and after compression test.
