# Supplementary material for: A Dual-Modified Chitosan-Derived Silica Composite Aerogel with Simultaneous Improvement of Mechanical, Flame Retardancy, and Thermal Insulation Properties
Source: Polymers (Basel). 2025 Nov 27;17(23):3162. doi: 10.3390/polym17233162 (PMC12693858; doi:10.3390/polym17233162)
Supplement: Supplementary file 1 [file polymers-17-03162-s001.zip › polymers-3974305-supplementary.pdf]

Supplementary Material

A dual-modified chitosan-derived silica composite aerogel with simultaneous improvement of mechanical, flame retardancy, and thermal insulation properties

Sicong Zhou[a], Ying Hou[b,c], Guifeng Xiang[d], Chuang Hu[b,c] *, Baisong Hu[a*], Yingxi Ji[a], Wei Zhang[a], Shaofeng Zhang [a]

[a] *School of Chemical Engineering and Technology, Hebei University of Technology, Tianjin 300130, China*

[b] *Chongqing Innovation Center, Beijing Institute of Technology, Chongqing 401120, China*

[c] *State Key Laboratory of Explosion Science and Safety Protection, Beijing Institute of Technology, Beijing 100081, China*

[d] *Hubei Aerospace Jianghe Chemical Co., Ltd, Yichang 444200, China*

TABLE OF CONTENT

S1. Tables

S2. Figures

* Corresponding author

E-mail: losaro@126.com (Chuang Hu); hubaisong@hebut.edu.cn (Baisong Hu).

S1. Tables

Table S1. The detailed elemental composition of PPA obtained from XPS spectra.

Element	C	O	N	P
Content (at%)	39.45	39.25	11.31	9.99

Table S2. Specific surface area (S_{BET}), pore diameter (d), and pore volume (V_{pore}) of aerogels.

**Sample**	CS	CA-2	CA/PPA-2.0
**Thermal conductivity (W/(m·K))**	0.048	0.039	0.042

Table S3. The mechanical property and MCC test data for the CA composite aerogel.

**Sample**	**Mechanical property**	**Fire behaviors under MCC**			
	Compressive strength at 90% compression (MPa)	$PHRR_m$ (W/g)	THR_m (kJ/g)	T_{PHRR} (°C)	HRC (J/(g·K))
CS	1.2 ±0.3	234.3 ±0.6	22.7 ±0.3	317.0 ±0.6	78.1 ±0.2
CA-1	2.7 ±0.3	31.0 ±0.3	9.7 ±0.2	166.8 ±0.5	10.3 ±0.3
CA-2	3.1 ±0.4	33.0 ±0.4	11.0 ±0.2	167.4 ±0.4	11.0 ±0.3
CA-3	1.1 ±0.2	38.4 ±0.4	12.4 ±0.2	164.7 ±0.4	12.8 ±0.4
CA-4	1.9 ±0.2	30.4 ±0.3	10.2 ±0.4	168.5 ±0.5	10.1 ±0.3
CA-5	1.2 ±0.2	38.3 ±0.4	11.9 ±0.2	168.6 ±0.5	12.8 ±0.4

Table S4. Flame retardancy results of CS-based aerogel composites in literatures and this work.

Flame retardants	Addition amount (wt%)	$\Delta PHRR_c$ (%)	ΔLOI (%)	References
**TCS-5.0**	5.0	32.4	39.5-29.0=10.5	[1]
**CCA 2**	2.0	58.4	29.5-25.5=4.0	[2]
**CA1.5P-AIP15**	15.0	22.3	41.1-26.8=14.3	[3]
**PCS-6**	6.0	92.2	80.0-38.0=42.0	[4]
**CSA-HGM-$Mg(OH)_2$**	16.7	68.3	50.8-28.0=22.8	[5]
**LBL6**	4.0	94.8	63.1-21.0=42.1	[6]
**CA/PPA-2.0**	2.0	96.0	82.5-32.3=50.2	This study

Notes: **TCS-5.0**: Phosphorus-containing aldehyde/ Chitosan aerogels; **CCA 2**: Chitosan Glutaraldehyde aerogels; **CA1.5P-AIP15**: Chitosan-aluminum/PVA (CAP) aerogels and aluminum isopropoxide (AIP); **PCS-6**: Phosphorylated chitosan; **CSA-HGM-$Mg(OH)_2$**: Incorporation of $Mg(OH)_2$ coated hollow glass microspheres (HGM) into chitosan (CSA) matrix and then cross-linking by glutaraldehyde; **LBL6**: Cellulose filaments/phytic acid/ chitosan aerogels; **$\Delta PHRR_c$ (%)**: $PHRR_c$ change after introducing flame retardants compared with neat polymer; **ΔLOI (%)**: LOI value change after introducing flame retardants compared with neat polymer. LOI values of neat CS is 32.3%.

Table S5. TGA data of the CA/PPA composite aerogels.

Sample	$T_{5\%}$ (°C)	T_d (°C)	MLR_{max} (%/°C)	CR (%)
CA-2	93	308	0.183	40.2
CA/PPA-0.5	92	297	0.188	39.1
CA/PPA-1.0	82	292	0.190	43.6
CA/PPA-2.0	92	287	0.192	54.5

Table S6. Mechanical properties of SA-based aerogel composites in literatures and this work.

Flame retardants	Compressive stress (MPa)	References
**WPCC/SA**	0.08	[7]
**SiC-SiO$_x$**	0.09	[8]
**Si-BN**	0.02	[9]
**CA/PPA-2.0**	0.11	This study

Notes: **WPCC/SA:** porous cellulose/silica aerogel; **SiC-SiO$_x$:** the laminated SiC-SiO$_x$ nanowire aerogel; **Si-BN:** boron nitride/silica aerogel.

S2. Figures

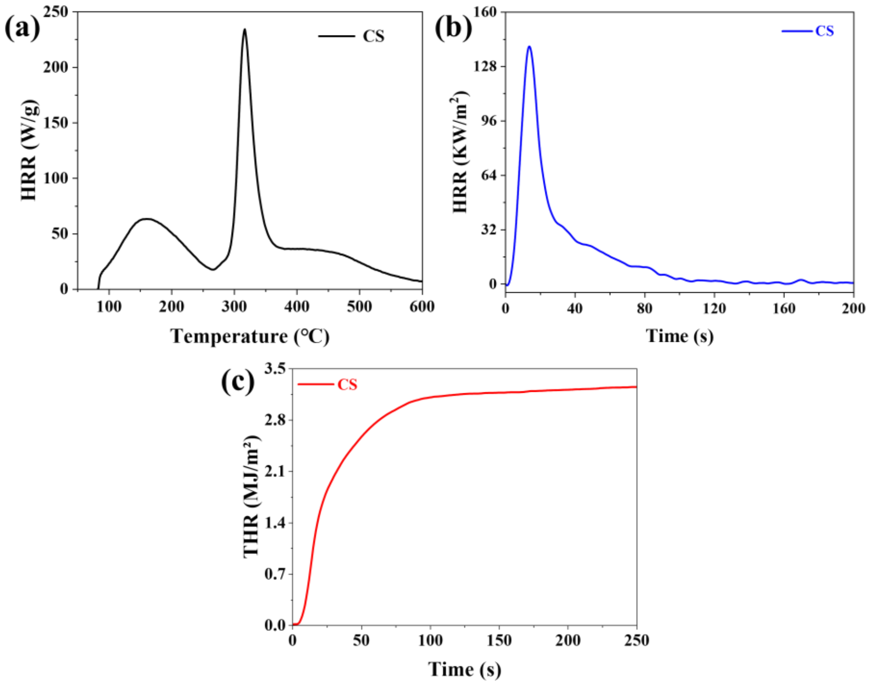

Figure S1. (a) HRR curves of neat CS cryogel under MCC test; (b) HRR and (c) THR curves of neat CS cryogel under CONE.

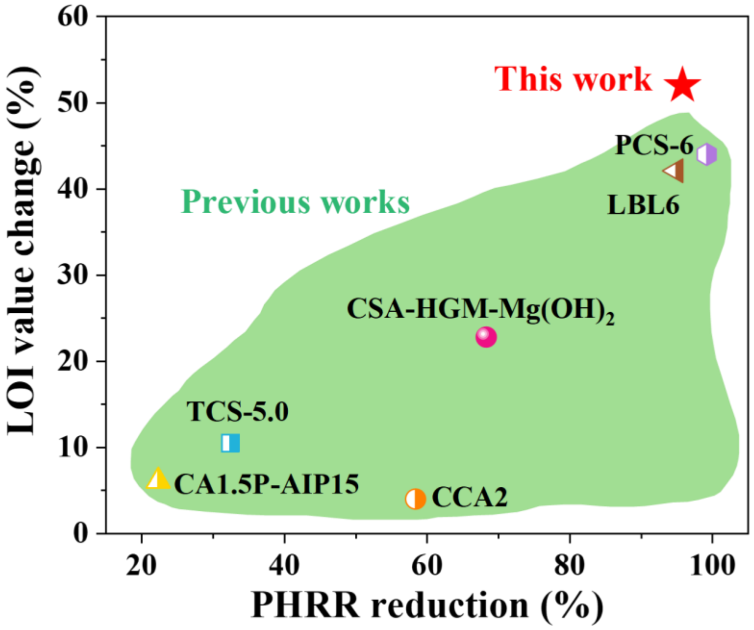

Figure S2. Comparison of flame retardancy for CS-based aerogel composites.

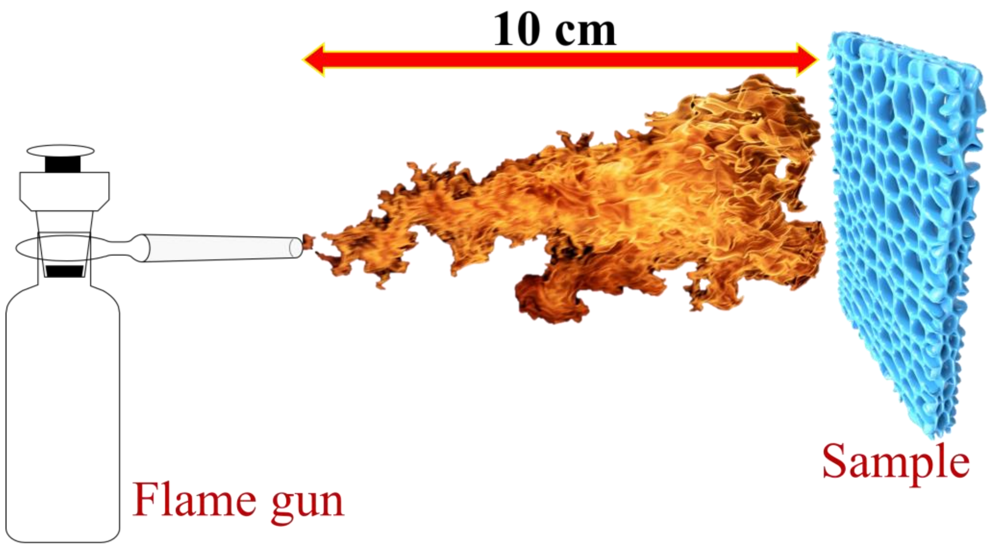

Figure S3. Schematic diagram of fire resistance evaluation experimental setup.

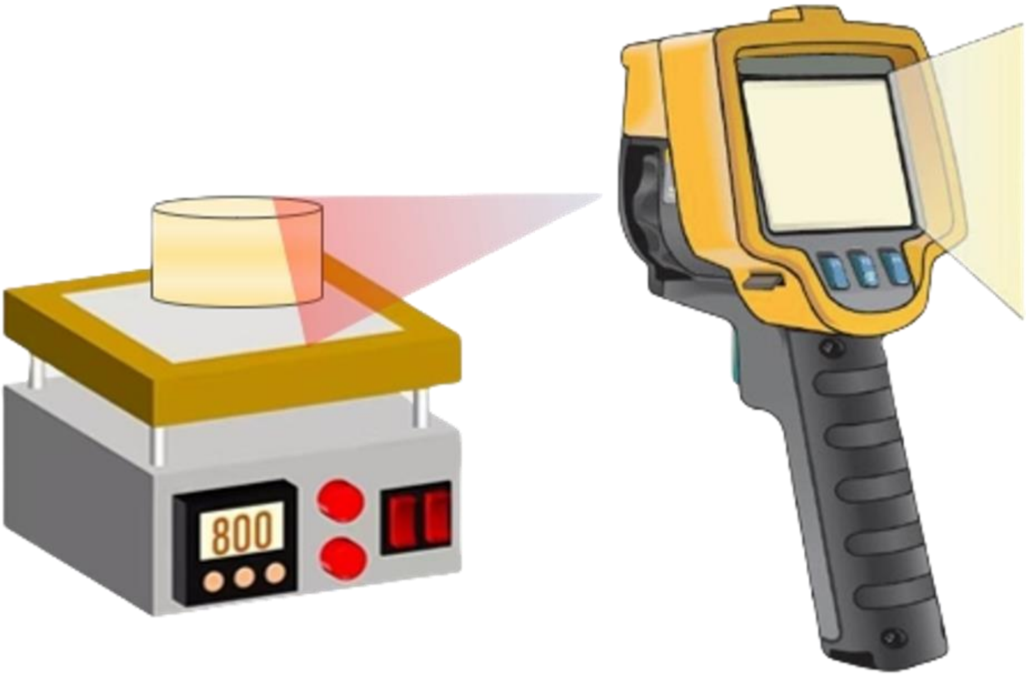

Figure S4. Schematic diagram of thermal infrared imaging experimental setup.

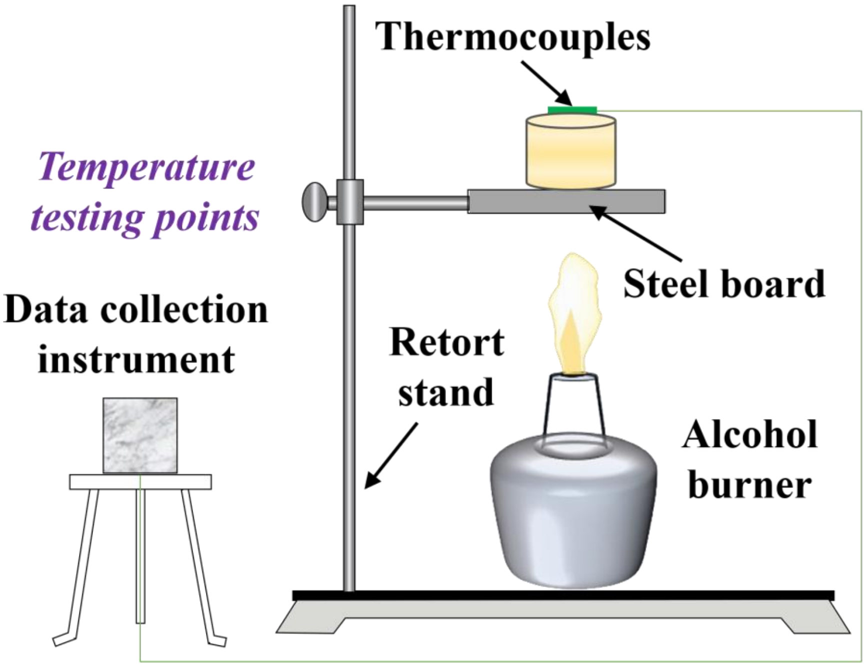

Figure S5. Schematic diagram of the alcohol burner experimental setup.

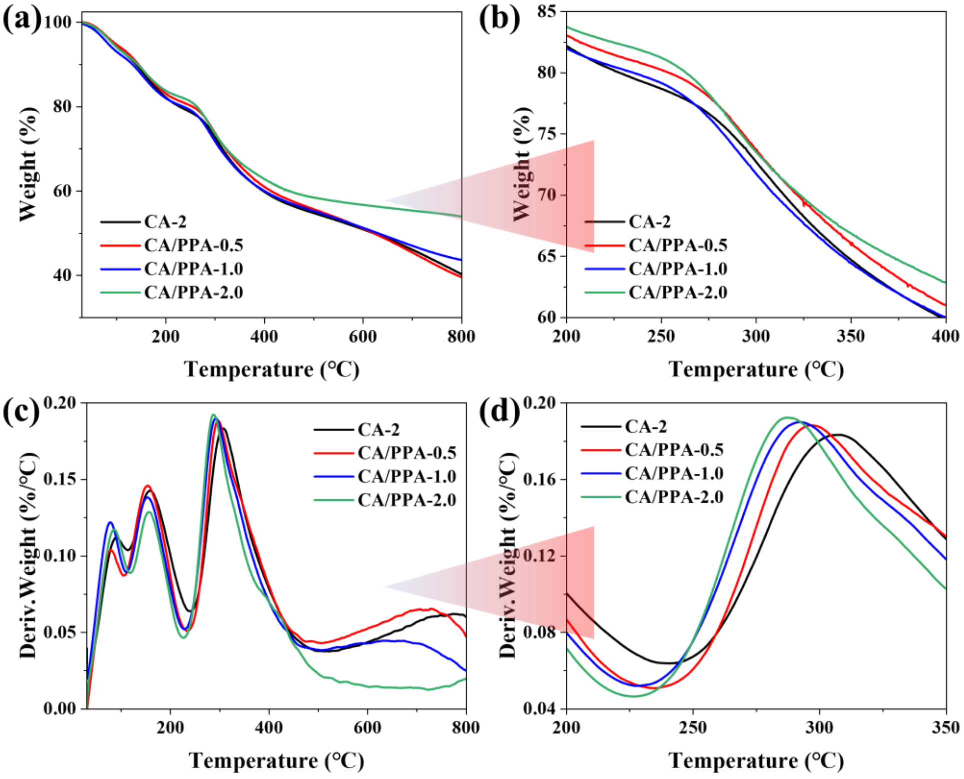

Figure S6. (a) and (b) TGA curves, and (c) and (d) DTG curves of the composite aerogels.

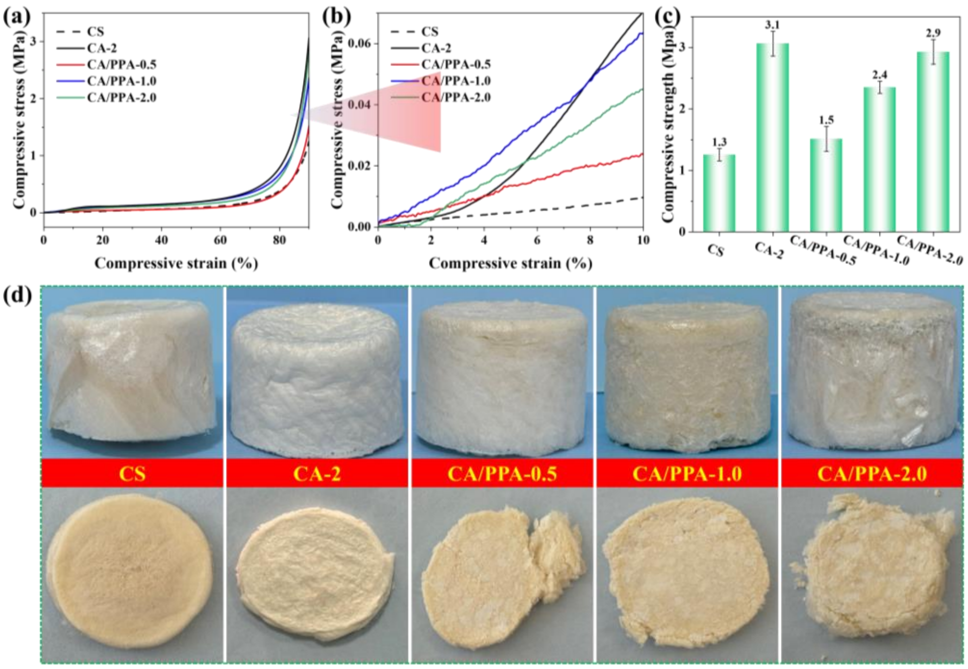

Figure S7. (a) and (b) Compressive stress curves of the CA/PPA composite and their (c) compressive strength; (d) Photographs of the CA/PPA composite before and after compression test.

References

[1] H. Niu, Z. Xiao, P. Zhang, W. Guo, Y. Hu, X. Wang, Flame retardant, heat insulating and hydrophobic chitosan-derived aerogels for the clean-up of hazardous chemicals, Science of The Total Environment 908 (2024) 168261.

[2] J. Chen, H. Xie, X. Lai, H. Li, J. Gao, X. Zeng, An ultrasensitive fire-warning chitosan/montmorillonite/carbon nanotube composite aerogel with high fire-resistance, Chemical Engineering Journal 399 (2020) 125729.

[3] Z. Yang, H. Li, G. Niu, J. Wang, D. Zhu, Poly(vinylalcohol)/chitosan-based high-strength, fire-retardant and smoke-suppressant composite aerogels incorporating aluminum species via freeze drying, Composites Part B: Engineering 219 (2021) 108919.

[4] H. Cui, N. Wu, X. Ma, F. Niu, Superior intrinsic flame-retardant phosphorylated chitosan aerogel as fully sustainable thermal insulation bio-based material, Polymer Degradation and Stability 207 (2023) 110213.

[5] Z. Zhu, Y. Niu, S. Wang, M. Su, Y. Long, H. Sun, W. Liang, A. Li, Magnesium hydroxide coated hollow glass microspheres/chitosan composite aerogels with excellent thermal insulation and flame retardancy, Journal of Colloid and Interface Science 612 (2022) 35-42.

[6] A. Varamesh, Y. Zhu, G. Hu, H. Wang, H. Rezania, Y. Li, Q. Lu, X. Ren, F. Jiang, S.L. Bryant, J. Hu, Fully biobased thermal insulating aerogels with superior fire-retardant and mechanical properties, Chemical Engineering Journal 495 (2024) 153587.

[7] X. Liu, T. Zhang, H. Xu, B. Li, G. Wei, X. Zhang, J. Zhang, Ionic liquid-mediated facile synthesis of porous cellulose/silica aerogel composites with improved thermal insulation, International Journal of Biological Macromolecules 307 (2025) 142418.

[8] L. Su, S. Jia, J. Ren, X. Lu, S.-W. Guo, P. Guo, Z. Cai, D. Lu, M. Niu, L. Zhuang, K. Peng, H. Wang, Strong yet flexible ceramic aerogel, Nature Communications 14(1) (2023) 7057.

[9] Y. Guo, S. Gu, R. Li, B. Xue, Q. Zhou, R. Yuan, L. Cong, Silicon-modified boron nitride aerogels with enhanced thermal stability for thermal insulation applications in high-temperature, Journal of Alloys and Compounds 1036 (2025) 181759.
